# Targeting ATR-CHK1 and ATM-CHK2 Axes in Pancreatic Cancer—A Comprehensive Review of Literature

**DOI:** 10.3390/ijms27031152

**Published:** 2026-01-23

**Authors:** Mateusz Kciuk, Katarzyna Wanke, Beata Marciniak, Damian Kołat, Marta Aleksandrowicz, Somdutt Mujwar, Tarik Ainane, Renata Kontek

**Affiliations:** 1 Department of Molecular Biotechnology and Genetics, University of Lodz, 90-237 Lodz, Polanddamian.kolat@umed.lodz.pl (D.K.);; 2 Department of Functional Genomics, Medical University of Lodz, 90-752 Lodz, Poland; 3 Laboratory of Preclinical Research and Environmental Agents, Mossakowski Medical Research Institute, Polish Academy of Sciences, 5 A. Pawińskiego Street, 02-106 Warsaw, Poland; 4 Chitkara College of Pharmacy, Chitkara University, Rajpura 140401, Punjab, India; 5 Superior School of Technology of Khenifra, University of Sultan Moulay Slimane, P.O. Box 170, Khenifra 54000, Morocco

**Keywords:** cell cycle checkpoint, combination regimens, DNA damage, DNA damage response (DDR), immunotherapy, pancreatic ductal adenocarcinoma (PDAC), precision medicine

## Abstract

Pancreatic cancer (PC) remains a highly lethal malignancy with limited treatment options and poor survival. Targeting DNA damage response (DDR) pathways has emerged as a promising therapeutic strategy, particularly the ATR-CHK1 and ATM-CHK2 axes. Preclinical studies demonstrate that ATR inhibition disrupts replication stress tolerance, impairs homologous recombination, and disables checkpoint control, enhancing cytotoxicity from standard therapies including gemcitabine, FOLFIRINOX, fluoropyrimidines, and radiotherapy. Synergistic effects have also been observed with other DDR-targeted agents, such as PARP and WEE1 inhibitors. Genomic contexts, including *ATM* deficiency, *ARID1A* alterations, and oncogene-driven replication stress, refine therapeutic sensitivity, supporting precision patient stratification. Early-phase clinical trials of ATR inhibitors (ART0380, AZD6738, BBI-355) alone or in combination show promising safety, tolerability, and preliminary efficacy. In this review, we summarize current literature on targeting the ATM-CHK2 and ATR-CHK1 pathways in PC, highlighting preclinical evidence, clinical developments, and strategies for biomarker-driven, precision oncology approaches.

## 1. Introduction

Pancreatic cancer remains one of the most lethal malignancies worldwide, currently ranking as the seventh leading cause of cancer-related mortality, with an estimated 505,752 deaths reported in 2021. Although it constitutes a relatively small proportion of overall cancer incidence, its mortality burden is disproportionately high due to the typically asymptomatic nature of early disease and frequent diagnosis at advanced stages. Global incidence has shown a marked upward trend over recent decades, with annual cases increasing from approximately 207,905 in 1990 to over 508,000 in 2021. This rise has been accompanied by an increase in the age-standardized incidence rate (ASIR) from 5.47 to 5.96 per 100,000 population during the same period. Similarly, the global burden of disease, measured in disability-adjusted life years (DALYs), more than doubled, rising from 5.21 million to 11.32 million [1]. Europe accounts for nearly 29% of global cases, with the highest incidence and mortality rates observed in high-income regions, including North America, Western Europe, and Australia. These trends are largely attributed to population aging and the growing prevalence of modifiable risk factors such as tobacco use, elevated body mass index (BMI), diabetes mellitus, and excessive alcohol consumption. Non-modifiable risk factors, including advancing age, inherited cancer predisposition syndromes, and pathogenic germline mutations, also play a significant role, with familial and hereditary factors estimated to account for approximately 10% of cases. Although advanced imaging modalities such as endoscopic ultrasound, magnetic resonance imaging, and computed tomography offer high sensitivity for lesion detection in high-risk individuals, their limited specificity, high cost, and invasive nature render them unsuitable for widespread screening in the general population. As a result, current early detection strategies are primarily limited to individuals with a strong family history or known genetic predisposition [2]. Despite improvements in diagnostic technologies and clinical management, the global five-year survival rate remains approximately 9%, highlighting the urgent need for improved prevention, early detection, and tailored public health interventions. While predictive models suggest a potential decline in ASIR and age-standardized mortality rate (ASMR) by 2050 in regions implementing effective control strategies, the global burden of pancreatic cancer is expected to remain substantial in the absence of comprehensive efforts to mitigate both modifiable risk factors and screening limitations [1,2,3,4].

**Figure 1:**
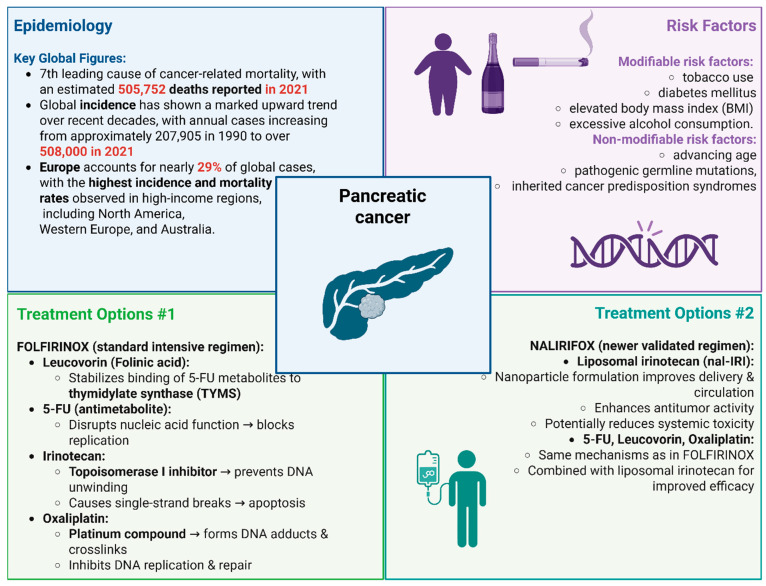
Pancreatic cancer: epidemiology, risk factors and treatment options. Created in BioRender. Kciuk, M. (2025) https://BioRender.com/slo07o7 (accessed on 30 December 2025).

Most patients present with unresectable or metastatic disease, making systemic therapy the cornerstone of treatment in the majority of cases. First-line systemic therapies for advanced disease currently include folinic acid (leucovorin; FOL), 5-fluorouracil (5-FU), irinotecan (IRI), oxaliplatin (OXA)—FOLFIRINOX, gemcitabine plus nab-paclitaxel, and the recently validated liposomal irinotecan (nal-IRI), FOL, 5-FU, and OX(A)—NALIRIFOX regimen. FOLFIRINOX is an intensive combination chemotherapy regimen that has become a cornerstone in the treatment of advanced pancreatic ductal adenocarcinoma (PDAC). Folinic acid (leucovorin), a vitamin B derivative, enhances the cytotoxic effect of 5-FU by stabilizing the binding of its active metabolites to the thymidylate synthase enzyme (TYMS), thereby prolonging its DNA synthesis-inhibiting activity. 5-FU, a pyrimidine analog and antimetabolite, is incorporated into RNA and DNA, ultimately disrupting nucleic acid function and cell replication. IRI, a topoisomerase I inhibitor, interferes with DNA unwinding during replication, leading to DNA strand breaks and cell death. OXA, a third-generation platinum compound, forms DNA adducts and cross-links, impairing DNA replication and repair mechanisms. In contrast, NALIRIFOX consists of four agents: nal-IRI, 5-FU, leucovorin, and OXA. Liposomal IRI (nal-IRI) allows for improved drug delivery and prolonged circulation time compared to conventional IRI, enhancing its antitumor activity while potentially reducing systemic toxicity [5,6,7,8]. Figure 1 displays epidemiological data and treatment options for pancreatic cancer.

A promising area of therapeutic development lies in the exploitation of deficiencies in the DNA damage response (DDR) pathway. A subset of PDAC tumors, particularly those harboring germline or somatic mutations in breast cancer type 1/2 susceptibility protein (*BRCA1/2*) or partner and localizer of BRCA2 (*PALB2*), exhibit homologous recombination deficiency (HRD), rendering them susceptible to DNA-damaging agents and DDR-targeted therapies [9,10,11]. In this context, poly (ADP-ribose) polymerase (PARP) inhibitors, such as olaparib, have demonstrated clinical benefit, with this drug receiving regulatory approval for maintenance therapy in germline *BRCA*-mutated metastatic PDAC following platinum-based chemotherapy [12,13,14,15,16].

Beyond *BRCA* mutations, efforts are underway to expand the therapeutic reach of DDR inhibition to tumors with other molecular vulnerabilities, including ataxia-telangiectasia mutated kinase (*ATM*) or checkpoint kinase 2 (*CHK2*) alterations and ataxia telangiectasia and rad3-related (*ATR*) and checkpoint kinase 1 (*CHK1*) mutations. Moreover, the integration of DDR-targeted agents with cytotoxic chemotherapy or immunotherapy is being actively explored to enhance efficacy and overcome resistance mechanisms. Despite the current limitations, namely, the relatively small proportion of patients eligible for such targeted approaches, DDR pathway inhibitors represent a rational and biologically grounded strategy with the potential to shift the treatment paradigm in molecularly selected subgroups of pancreatic cancer.

## 2. ATR/CHK1 Axis in DDR

**Figure 2:**
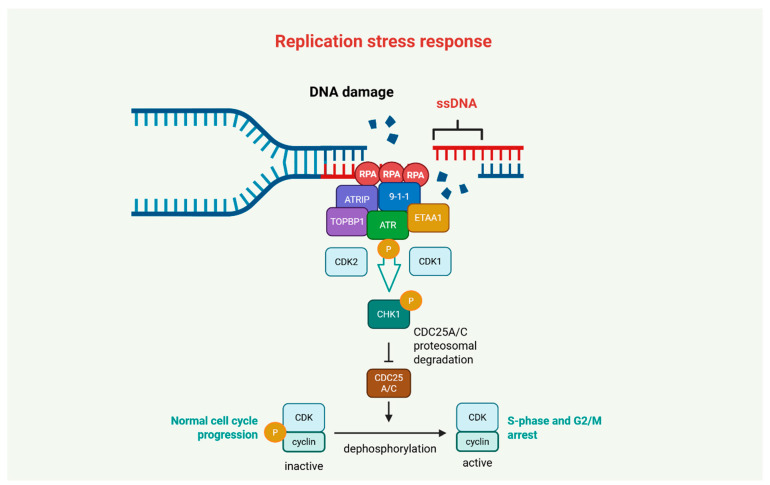
Involvement of ataxia telangiectasia and Rad3-related (ATR) kinase and checkpoint kinase 1 (CHK1) axis in replication stress response. ATR and CHK1 enforce cell cycle arrest in response to replication stress (RS) and DNA damage. RS causes single-stranded DNA (ssDNA) accumulation, which is coated by replication protein A (RPA), recruiting ATR via ATR-interacting protein (ATRIP). ATR phosphorylates RPA32 at Ser-33, followed by cyclin-dependent kinases 1/2 (CDK1/2) phosphorylation at Ser-23 and Ser-29. ATR activation is amplified by topoisomerase 2-binding protein 1 (TOPBP1), the RAD9-RAD1-HUS1 (9-1-1) complex, and Ewing’s tumor-associated antigen 1 (ETAA1). Activated CHK1 phosphorylates cell division cycle 25 (CDC25) phosphatases (CDC25A and CDC25C), promoting their degradation, inhibiting CDKs, and blocking G1/S and G2/M progression to allow DNA repair. Created in BioRender. Kciuk, M. (2025) https://BioRender.com/hwv0cps (accessed on 30 December 2025).

The ATR-CHK1 signaling axis plays a central role in the cellular response to replication stress (RS), a hallmark of cancer that contributes to genomic instability and tumor progression. ATR is activated primarily in response to the accumulation of single-stranded DNA (ssDNA) at stalled replication forks, which are rapidly coated by replication protein A (RPA). ATR is recruited and activated through its binding partner ATR-interacting protein (ATRIP), with further stimulation provided by co-factors such as DNA topoisomerase 2-binding protein 1 (TOPBP1), the RAD9-RAD1-HUS1 (9-1-1) complex [17,18,19], and the recently identified Ewing tumor-associated antigen 1 (ETAA1) [20,21,22,23]. ATR subsequently phosphorylates numerous substrates, including CHK1, which mediates downstream cell cycle arrest and replication fork stabilization. Full CHK1 activation is achieved through phosphorylation at Ser-317 and Ser-345 by ATR [24,25,26], with additional cyclin-dependent kinases 1/2 (CDK1/2)-mediated phosphorylation at Ser-286 and Ser-301 enhancing its activity [27]. Activated CHK1 enforces cell cycle checkpoints by targeting cell division control 25 (CDC25) phosphatases for degradation, thereby preventing activation of CDK-cyclin complexes and halting cell cycle progression at G1/S and G2/M cell cycle restriction points. This checkpoint enforcement allows time for DNA repair and preservation of genome integrity [28,29] (Figure 2). Importantly, cancer cells, particularly those driven by oncogene-induced RS (e.g., *RAS*, *MYC*, or cyclin E overexpression), become increasingly reliant on the ATR-CHK1 axis for survival, rendering this pathway a promising therapeutic target [30].

## 3. ATM/CHK2 Axis in DDR

The ATM-CHK2 signaling axis represents a fundamental pathway within the DNA damage DDR network, primarily activated in response to DNA double-strand breaks (DSBs). Upon DSB induction, the MRE11-RAD50-NBS1 (MRN) complex detects and binds DNA ends, facilitating the recruitment and activation of ATM kinase [31,32,33,34]. ATM undergoes autophosphorylation and dissociation of inactive dimers to active monomers at the damage sites and triggers a robust phosphorylation cascade targeting a wide range of substrates, including mediator of DNA damage checkpoint protein 1 (MDC1), nibrin (NBS1), TP53-binding protein 1 (53BP1), BRCA1, and the histone variant H2AX [35,36,37].

Phosphorylation of H2AX at serine 139 by both the ATM and ATR kinases initiates γH2AX foci formation [38,39,40] that acts as a platform for MDC1 recruitment and amplification of the DNA damage signal [41,42,43] through downstream E3 ubiquitin ligases such as RNF8, RNF168, and RNF4 [44,45,46,47]. These events orchestrate the accumulation of critical repair proteins, including 53BP1 [48,49,50] and BRCA1 [51,52], at the sites of damage, thereby influencing the balance between non-homologous end joining (NHEJ) and homologous recombination (HR) repair [53,54,55].

Among ATM’s critical downstream targets is CHK2, which is phosphorylated at threonine 68, promoting its dimerization, autophosphorylation, and full activation [56,57,58]. Activated CHK2 phosphorylates various substrates, including CDC25A and CDC25C phosphatases, leading to their degradation or sequestration and cell cycle arrest in G1/S or G2/M phases, thereby allowing time for DNA repair [59,60,61,62,63].

Additionally, CHK2-mediated phosphorylation of BRCA1 at serine 988 is essential for efficient HR, underscoring the integration of ATM-CHK2 signaling with HR repair pathways [64]. BRCA1 itself functions as a scaffolding molecule, facilitating the activity of ATM and ATR on their respective substrates and coordinating the repair of DNA through DNA repair protein RAD51 homolog 1 (RAD51) recruitment and HR [65,66,67,68,69,70].

Furthermore, the phosphorylation and recruitment dynamics of DDR components are modulated by CDKs, whose activity not only influences BRCA1 [71,72,73] and 53BP1 [74,75] localization but also affects chromatin remodeling via RNF4, RNF8, RNF168-mediated ubiquitination of H2A/γH2AX affecting DNA repair dynamics [44,76,77].

**Figure 3:**
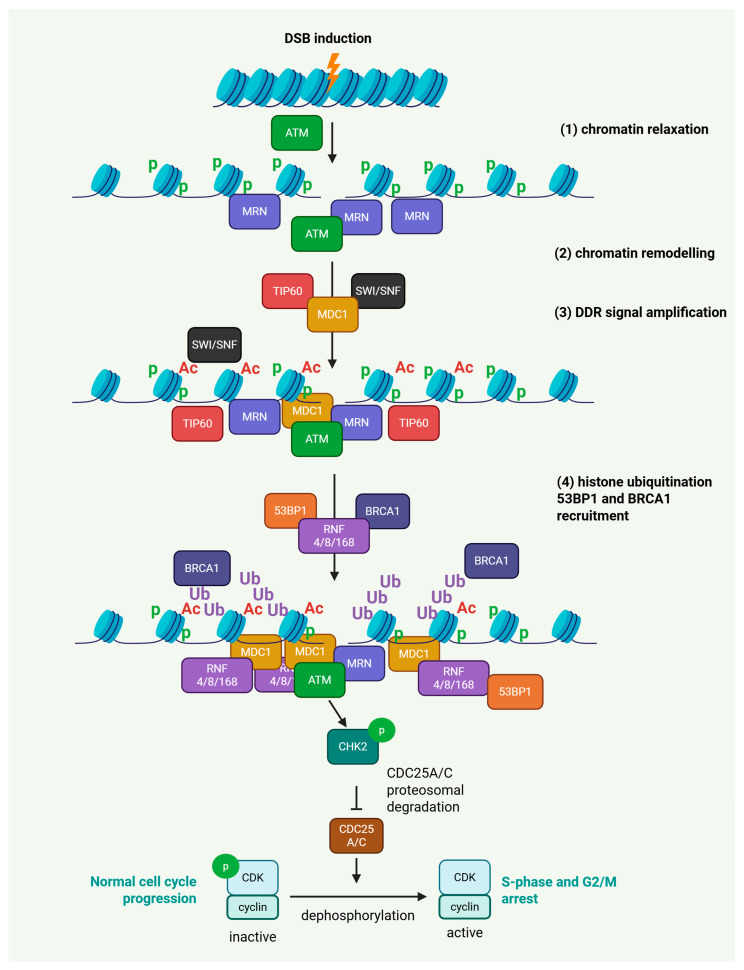
Ataxia-telangiectasia mutated kinase (ATM)-checkpoint kinase 2 (CHK2) signaling of DNA double-strand breaks (DSBs) in eukaryotic cells in the context of chromatin dynamics. DNA damage induces dynamic chromatin remodeling that governs the accessibility and efficiency of DNA damage response (DDR) signaling. Following DSBs occurrence, mediator of DNA damage checkpoint protein 1 (MDC1) binds phosphorylated (p) histone H2AX (γH2AX) and functions as a central scaffold to amplify DDR signaling by recruiting ubiquitin ligases Ring Finger Protein 8 (RNF8), Ring Finger Protein 168 (RNF168), and Ring Finger Protein 4 (RNF4). These factors promote ubiquitination (Ub) of histone H2A and γH2AX, facilitating the ordered recruitment of downstream repair proteins such as BRCA1 and p53-binding protein 1 (53BP1) and shaping repair pathway choice. Nucleosomal compaction initially restricts access to damaged DNA; therefore, one of the earliest DDR events is chromatin relaxation, which enables efficient recruitment of sensors and mediators. This relaxation is driven by histone acetyltransferases Tat-interactive protein 60 kDa (TIP60) and CREB-binding protein (CBP) and its paralog p300 (CBP/p300), which acetylate histone tails (Ac), as well as ATP-dependent SWItch/Sucrose Non-Fermentable (SWI/SNF) chromatin remodeling complexes that reposition nucleosomes. During repair, chromatin is maintained in a permissive state to allow lesion processing, ATM-CHK2 signaling followed by gradual re-compaction through histone deacetylation and restoration of higher-order chromatin structure to preserve genome stability and epigenetic integrity. Created in BioRender. Kciuk, M. (2026) https://BioRender.com/p1xy49x (accessed on 30 December 2025).

Importantly, the chromatin environment itself critically determines the accessibility and efficiency of DDR signaling. Nucleosomal compaction poses a physical barrier to repair factors, and one of the earliest events following DNA damage is chromatin relaxation, which facilitates recruitment of sensors and mediators to the lesion. This relaxation is promoted by histone acetyltransferases such as Tat-interactive protein 60 kDa (TIP60) and CREB-binding protein (CBP) and its paralog p300 (CBP/p300), which acetylate histone tails to weaken nucleosome–DNA interactions [78,79,80,81,82,83], as well as by ATP-dependent remodeling complexes like SWItch/Sucrose Non-Fermentable (SWI/SNF), which reposition nucleosomes to create open chromatin domains [84,85,86]. Once repair is initiated, chromatin must be locally maintained in a permissive state to allow efficient processing of DNA lesions. Following repair completion, compaction is gradually restored to preserve genome stability and epigenetic integrity, in part through deacetylation and re-establishment of higher-order chromatin structure. Thus, the interplay between histone modifications, nucleosome remodeling, and chromatin re-compaction ensures a dynamic but tightly regulated chromatin response that coordinates DDR signaling with faithful repair outcomes [87,88,89] (Figure 3).

## 4. Downstream Events in ATR-CHK1 and ATM-CHK2 Axis

Following activation of the ATM-CHK2 and ATR-CHK1 signaling axes, a series of coordinated downstream events are initiated that determine the fate of the cell, particularly through the activation and stabilization of the cellular tumor antigen P53 (TP53). Both ATM [90,91,92,93,94,95] and ATR [96,97,98,99,100], as well as their effector kinases CHK1 [101,102,103,104] and CHK2 [101,102,105,106], directly phosphorylate TP53 at multiple serine residues, a modification that leads to the dissociation of TP53 from its negative regulator, the E3 ubiquitin ligase—mouse double minute 2 homolog (MDM2) [107,108,109,110]. This stabilization enables TP53 to form a functional tetrameric transcription factor that regulates the expression of critical genes involved in cell cycle arrest and apoptosis [111,112,113]. One of the principal TP53 transcriptional targets is cyclin-dependent kinase inhibitor 1A (P21), which binds to and inhibits cyclin-CDK complexes, particularly cyclin E-CDK2, thereby preventing the phosphorylation of retinoblastoma protein (pRB) and blocking the G1-to-S phase transition of the cell cycle. Additionally, P21 can modulate interactions between proliferating cell nuclear antigen (PCNA) and DNA repair proteins, contributing to the coordination of DNA repair [114,115,116,117,118]. Simultaneously, TP53 upregulates pro-apoptotic genes such as BCL2 associated X, apoptosis regulator (*BAX*) and p53 up-regulated modulator of apoptosis (*PUMA*). BAX, in conjunction with BAK, oligomerizes to form pores in the mitochondrial outer membrane, leading to the release of cytochrome c and subsequent activation of the intrinsic apoptotic pathway. PUMA further amplifies this response by antagonizing anti-apoptotic proteins such as B-cell lymphoma 2 (BCL-2) and B-cell lymphoma-extra large (BCL-XL), thus facilitating the mitochondrial permeabilization process [119,120,121,122].

### 4.1. Single-Strand DNA Break Repair (SSBR)

Single-strand breaks (SSBs) represent the most frequent form of DNA lesions and, if not efficiently repaired, pose a critical threat to genomic stability, particularly during DNA replication. The cellular response to SSBs is orchestrated through both ATR–CHK1 and ATM–CHK2 axes. As previously mentioned, the ATR–CHK1 pathway is primarily activated when replication forks encounter unrepaired SSBs, leading to the generation of RPA-coated ssDNA. ATR, recruited by ATRIP to these ssDNA regions, phosphorylates and activates CHK1, thereby stabilizing stalled forks, enforcing the intra-S phase checkpoint, and coordinating single-strand DNA break repair (SSBR) [123]. Mechanistically, resolution of SSBs is initiated by PARP1, which rapidly detects and binds to DNA termini at the break site. Activated PARP1 catalyzes the addition of poly(ADP-ribose) (PAR) chains that serve as a platform for the recruitment of scaffold proteins such as X-ray repair cross-complementing protein 1 (XRCC1) [124,125,126,127]. XRCC1, in turn, assembles and stabilizes a multiprotein repair complex by recruiting and coordinating the activities of polynucleotide kinase/phosphatase (PNKP), aprataxin (APTX), DNA polymerase β (POLB), and DNA ligase III (LIG3). This ensemble ensures end processing, gap filling, and final ligation of the break as reviewed by other authors [128,129,130].

### 4.2. Double-Strand DNA Break Repair (DSBR)

Conversely, persistent or unresolved SSBs encountered by replication forks frequently collapse into DSBs, which are sensed by the MRN complex and signal through ATM. Activated ATM phosphorylates CHK2, inducing cell-cycle arrest at the G1/S or G2/M transition and facilitating double-strand DNA break repair (DSBR) [131,132,133]. Mechanistically, repair of replication-associated one-ended DSBs is primarily accomplished by HR, initiated by MRN-mediated DNA end resection to generate 3′ ssDNA overhangs, which are subsequently coated by RPA and replaced by RAD51 filaments to facilitate strand invasion and homology search [134,135,136]. In situations where HR is compromised or in non-S-phase contexts, ATM signaling can also channel repair into NHEJ, which directly ligates DNA ends with the assistance of KU70/80 proteins, DNA-dependent protein kinase catalytic subunit (DNA-PKcs), and ligase IV (LIG4). The ATM–CHK2 axis, therefore, acts as a safeguard mechanism, converting collapsed forks into substrates for DSB repair while simultaneously enforcing global cell-cycle control and promoting repair pathway choice depending on the cell-cycle phase and chromatin context [137,138,139,140].

## 5. Preclinical Studies

### 5.1. ATR Inhibitors

Inhibition of ATR has been proposed as a potential means to sensitize tumor cells to DNA-damaging agents and radiotherapy. Fokas et al. assessed the radiosensitizing activity of VE-821, a potent ATR inhibitor, both in vitro and in vivo. ATR activity was monitored via CHK1 phosphorylation, DNA damage was evaluated using γH2AX and 53BP1 foci formation, while HR involvement in the DNA repair processes was assessed through RAD51 foci formation. In vitro, VE-822 disrupted cell-cycle checkpoint maintenance, increased levels of DNA damage, and inhibited HR in irradiated pancreatic cancer cells, leading to decreased clonogenic survival. Notably, normal cells were largely unaffected. In vivo, VE-822 significantly prolonged tumor growth delay in pancreatic cancer xenografts following radiation or gemcitabine-based chemoradiation, without exacerbating intestinal or other normal tissue toxicity. These findings suggested that ATR inhibition with VE-822 may enhance the therapeutic potential of radiochemotherapy in PDAC, representing a promising strategy to improve clinical outcomes while minimizing collateral tissue damage [141].

In contrast, Bruciamacchie et al. evaluated the effects of the combination of FOLFIRINOX with VE-822 in PDAC spheroid and organoid models. The drug combination exhibited strong synergistic effects across multiple cell lines regardless of *BRCA1*, *BRCA2*, or *ATM* mutation status, as well as showed efficacy in models resistant to standard chemotherapy. Mechanistic analyses revealed enhanced DNA damage, suppression of DDR signaling, and increased proportions of apoptotic cells when combination treatment was employed. Importantly, in patient-derived xenografts and immunocompetent orthotopic KPC mouse models (K: Kras^^^LSL-G12D—a conditional allele that activates the oncogenic Kras^^^G12D mutation; P: Trp53^^^LSL-R172H—a conditional allele introducing a mutant p53 (R172H), a common tumor suppressor mutation in PDAC; C: Pdx1-Cre or Ptf1a-Cre—a pancreas-specific Cre recombinase that drives expression of the mutant alleles in pancreatic tissue), the combination suppressed tumor growth more effectively than FOLFIRINOX alone. This was accompanied by remodeling of the tumor microenvironment, including a reduction in fibroblast activation protein-positive cancer-associated fibroblasts (CAFs) and increased infiltration of antitumor immune populations. Collectively, these results indicated that ATR inhibition can potentiate FOLFIRINOX efficacy and overcome resistance mechanisms in PDAC, supporting further investigation of this therapeutic strategy [142].

In another study, the ATR-selective inhibitor AZ20 was evaluated in five pancreatic cancer cell lines, both as monotherapy and in combination with gemcitabine. AZ20 alone suppressed cell growth in a dose-dependent manner but induced only limited cytotoxic effects. Consistent with ATR inhibition, AZ20 reduced CHK1 phosphorylation at Ser345 and triggered DNA damage accumulation with S-phase and G2/M cell-cycle arrest, irrespective of *TP53* mutational status of the examined pancreatic cells. Notably, combining AZ20 with gemcitabine produced synergistic growth inhibition and enhanced cell death across all models tested. Mechanistically, ATR inhibition amplified gemcitabine-induced DNA damage and markedly suppressed gemcitabine-driven upregulation of the ribonucleotide reductase M2 subunit (RRM2) [143].

In contrast, Wallez et al. evaluated AZD6738 as an ATR inhibitor in combination with gemcitabine in preclinical PDAC models. In vitro, AZD6738 suppressed gemcitabine-induced CHK1 activation, prevented cell-cycle arrest, and blocked accumulation of RRM2, leading to pronounced RS only under combination treatment. Synergistic growth inhibition was demonstrated across multiple mouse and human PDAC cell lines, with clonogenic survival assays confirming near-complete abrogation of colony formation at doses ineffective as monotherapies. In vivo, the combination was well tolerated and produced significant tumor regression in both subcutaneous allografts and a subset of autochthonous KPC mouse tumors, ultimately extending survival. These findings provided a strong preclinical rationale that ATR inhibition can potentiate gemcitabine activity by disabling checkpoint-mediated resistance, supporting clinical evaluation of AZD6738 plus gemcitabine in patients with PDAC [144].

In another study, Dunlop et al. explored whether *ATM* loss could predict sensitivity to the combination of the ATR inhibitor AZD6738 and gemcitabine. Using pharmacological inhibition, small interfering RNA (siRNA) depletion, and clustered regularly interspaced short palindromic repeats (CRISPR) knockout approaches, investigators assessed how *ATM* targeting influenced PDAC cell responses to ATR inhibitor and gemcitabine. Complete *ATM* loss, achieved through inhibition or knockout, but not partial depletion, markedly sensitised PDAC cells to the combination. Mechanistically, the combination of drugs induced replication catastrophe, enhanced in *ATM*-deficient cells. In vivo studies demonstrated that while the therapeutic combination produced only growth delay in *ATM*–wild-type xenografts, it induced tumor regression in *ATM*-knockout xenografts. These findings suggested that *ATM* loss amplifies RS-mediated cell death induced by AZD6728 and gemcitabine combination and may serve as a predictive biomarker of therapeutic response. Careful assessment of *ATM* status, particularly distinguishing between *ATM*-low and *ATM*-null tumors, could therefore be critical for the success of clinical trials evaluating this therapeutic combination [145].

Höfer et al. performed a large-scale phenotypic screen of 13 PDAC cell lines treated with gemcitabine in combination with 146 clinically relevant inhibitors and identified the ATR kinase inhibitor elimusertib as a particularly strong synergistic partner in the majority of models. Furthermore, mechanistic insights were provided by dose-dependent phosphoproteomic profiling of four ATR inhibitors (berzosertib, AZD6738, elimusertib, and gartisertib) in the context of gemcitabine-induced DNA damage, which revealed a potent suppression of DDR signaling, including blockade of CHK1 phosphorylation at Ser468. Although CHK1 activation is classically monitored via phosphorylation at Ser317 and Ser345, these sites are subject to redundancy and partial compensation by ATM and other upstream kinases under conditions of replication stress. In contrast, Ser468 resides within a canonical SQ motif and emerged from the phosphoproteomic dataset as a highly ATR-selective, dose-responsive site that was robustly induced by gemcitabine (~18-fold) and fully suppressed by all four ATR inhibitors. These findings established ATR inhibition as a robust enhancer of gemcitabine efficacy, providing both a mechanistic rationale and a molecular resource for the rational design of combinational treatments in PDAC [146].

To further delineate the impact of ATM deficiency on genome integrity, conditional ATM deletion was examined in a genetically engineered mouse model of PDAC. Loss of ATM resulted in elevated mitotic defects, recurrent genomic rearrangements, and impaired DNA integrity checkpoint regulation, closely resembling features of human PDAC. Given these alterations, a synthetic lethality-based therapeutic approach was explored. ATM-deficient PDAC cells displayed heightened sensitivity to PARP inhibition with olaparib and to ATR inhibitor VE-822, both in vitro and in vivo, associated with genotype-selective induction of apoptosis [147].

Furthermore, Morimoto et al. examined the effects of combining S-1 (chemotherapeutic agents consisting of tegafur—a prodrug of 5-FU, gimeracil—a dihydropyrimidine dehydrogenase inhibitor that prevents the rapid degradation of 5-FU, thereby prolonging its plasma half-life and enhancing its antitumor activity and oteracil potassium—which reduces phosphorylation of 5-FU in the gastrointestinal tract and helps limit toxicity) with AZD6738 using four pancreatic cancer cell lines (BxPC-3, SUIT-2, PANC-1, and MIA PaCa-2). MTT assays revealed that the drug combination synergistically suppressed cell proliferation, while flow cytometry demonstrated a significant increase in apoptosis relative to either agent used standalone. Mechanistic studies indicated that AZD6738 blocked S-1-induced activation of ATR and CHK1, thereby disrupting DDR signaling. In vivo, xenograft experiments showed marked tumor growth inhibition, with mean tumor volumes substantially reduced in the combination group compared with single-agent groups (298 mm^3^ vs. 601 mm^3^ for S-1 and 580 mm^3^ for AZD6738 after weeks). Together, these findings suggested that dual targeting of ATR and DNA RS with AZD6738 and S-1 represents a promising strategy to enhance antitumor activity in pancreatic cancer [148].

Additionally, novel polymeric fluoropyrimidines, including F10 and CF10, have been developed to selectively generate the FdUMP while avoiding the toxic ribonucleotide metabolites associated with 5-FU. The investigators confirmed the superior potency of CF10, demonstrating a 308-fold increase in activity compared to 5-FU, with efficacy in the nanomolar range across primary patient-derived PDAC models. Mechanistic evaluation revealed that CF10 activity could be further enhanced by co-treatment with inhibitors of ATR and G2 checkpoint kinase (WEE1), key regulators of S- and G2-phase DNA damage checkpoints, implicating RS as a major driver of its cytotoxicity. Importantly, the addition of deoxynucleotides reversed CF10 activity, further supporting RS as an underlying mechanism. These findings indicate that CF10 not only holds promise as a more effective and less toxic alternative to 5-FU in PDAC therapy, but also that its efficacy may be potentiated through rational combinations with DNA damage checkpoint inhibitors [149].

Inhibitors of key checkpoint kinases, including WEE1, ATR, and CHK1, have been explored for their potential to enhance the cytotoxic effects of gemcitabine. Comparative analyses in pancreatic cancer-derived and osteosarcoma-derived cell lines demonstrated that all three checkpoint inhibitors increased gemcitabine sensitivity, with WEE1 inhibition producing the most pronounced effect. Mechanistic interrogation revealed that WEE1 inhibition not only abrogated its canonical role in cell-cycle control but also attenuated gemcitabine-induced ATR/CHK1 activation. This effect was shown to depend on CDK1/2 and Polo-like kinase 1 (PLK1), whose activation reduced claspin and C-terminal binding protein (CtBP) interacting protein (CtIP) levels, proteins involved in phosphorylation and activation of CHK1 by ATR in response to RS or DNA damage, and DNA end resection during HR, respectively. Collectively, these findings delineated a signaling cascade in which WEE1 inhibition disrupts ATR/CHK1 activity, intensifies RS, and enhances chemosensitivity to nucleoside analogs. These results positioned WEE1 as a particularly promising therapeutic target for combination strategies in PDAC [150].

#### 5.1.1. Combination of ATR and PARP-1/2 Inhibitors

The PARP-1/2 inhibition to enhance radiation-induced cytotoxicity in pancreatic adenocarcinoma has been evaluated both in vitro and in vivo. In pancreatic carcinoma cell lines, treatment with the PARP inhibitor ABT-888 in combination with radiation significantly increased cell death compared with either treatment alone, with dose enhancement factors ranging from 1.29 to 2.36 depending on ABT-888 concentration. While ABT-888 alone had minimal effect on caspase activity, combined treatment with radiation significantly increased apoptosis. Radiation-induced PARP activity was effectively inhibited by ABT-888, indicating interference with DDR pathways. In orthotopic xenograft models, co-treatment with ABT-888 and radiation extended tumor growth inhibition to 39 days and improved 60-day survival to 40%, compared with either monotherapy. The study suggested that ABT-888 potentiates radiation-induced DNA damage through inhibition of PAR protein polymerization, accompanied by feedback upregulation of PARP and ATM, highlighting their potential as predictive biomarkers [151].

A study by Hou et al. examined the role of PARP-1 in regulating proliferation and apoptosis in *TP53*-dependent pancreatic cancer. Using PanC-1 cells, PARP-1 expression was suppressed with olaparib, resulting in reduced cell proliferation and increased apoptosis relative to controls. Mechanistically, PARP-1 inhibition decreased pro–caspase-3 expression, enhanced caspase-3 activity, reduced BCL-2 protein levels, and increased TP53 expression. Notably, concomitant inhibition of ATM further amplified these effects: combined PARP-1 suppression and *ATM* inactivation markedly reduced cell viability, enhanced TP53 expression, increased caspase-3 activity, and suppressed BCL-2 expression compared with PARP-1 suppression alone. These findings indicated that targeting of PARP-1 induces apoptosis in pancreatic cancer cells through a TP53-dependent mechanism, which is intensified under *ATM*-deficient conditions [152].

While maintenance therapy with the PARP inhibitor olaparib has demonstrated improved progression-free survival in patients with germline *BRCA1/2* mutations following platinum-based induction, the applicability of this strategy to tumors with other DDR alterations was less clear. To address this, a preclinical placebo-controlled, three-arm study evaluated the efficacy of multi-DDR interference (mDDRi) as a maintenance therapy compared with continued FOLFIRINOX treatment, using orthotopically transplanted *ATM*-deficient PDAC cell lines. Survival analyses revealed a significant improvement in median overall survival (OS) in the mDDRi maintenance group relative to continuous FOLFIRINOX, which was accompanied by greater DNA-damage accumulation, enhanced disease control, and reduced metastatic dissemination. In vitro findings suggested that while FOLFIRINOX promoted the selection of invasive tumor subclones, subsequent mDDRi treatment eliminated these populations. Collectively, the study provided preclinical evidence that mDDRi may represent a promising maintenance strategy for *ATM*-deficient PDAC, extending the benefits of DDR-targeted therapy beyond germline *BRCA1/2*-mutant disease [153].

The majority of pancreatic cancers are HR-proficient and intrinsically resistant to PARP inhibitor monotherapy. To overcome this limitation, one study investigated whether combining olaparib with radiation and the ATR inhibitor AZD6738 could extend therapeutic efficacy to HR-proficient disease. The combination of olaparib and AZD6738 markedly enhanced radiosensitivity compared with either agent alone, independent of HR status. In HR-deficient models, low concentrations of olaparib provided radiosensitization through catalytic inhibition of PARP, whereas HR-proficient models required higher concentrations that induced PARP1-DNA complex formation. Importantly, Clustered Regularly Interspaced Short Palindromic Repeats–CRISPR-associated protein 9 (CRISPR-Cas9)-mediated PARP1 deletion eliminated the radiosensitizing effects of olaparib and abrogated the efficacy of the olaparib–AZD6738 combination, highlighting a critical role for PARP1-DNA complexes rather than catalytic inhibition in mediating therapeutic effect. Mechanistic analyses revealed that the triple combination of olaparib, radiation, and AZD6738 increased the prevalence of DSBs. DNA fiber assays further showed that high concentrations of olaparib accelerated replication fork progression, triggering ATR-mediated RS that was suppressed by AZD6738. In HR-proficient xenograft models, this combination therapy significantly delayed tumor growth compared with single or dual treatments. These findings suggested that PARP1-DNA complex formation is central to the therapeutic benefit of olaparib when combined with ATR inhibition and radiation, supporting clinical evaluation of this strategy for HR-proficient pancreatic cancers resistant to PARP inhibitor monotherapy [154].

TOPBP1 is one of the key regulators of DDR, and its overexpression has been implicated in tumorigenesis across multiple cancer types. Tang et al. examined the role of TOPBP1 in PDAC pathogenesis and its potential as a predictive biomarker for therapy. Analysis of PDAC cell lines, primary cells, and subcutaneous mouse models demonstrated that elevated *TOPBP1* expression correlated with higher histologic grade and poorer patient survival. Knockdown of *TOPBP1* enhanced the sensitivity of PDAC cells to the PARP inhibitor olaparib and improved therapeutic efficacy in vivo. Furthermore, combination treatment with olaparib and the ATR inhibitor AZD6738 induced TP53-dependent apoptosis by inhibiting ATR signaling while enhancing ATM pathway activity, significantly reducing cell viability. Notably, this combination therapy was most effective in PDAC models with high *TOPBP1* expression, suggesting that TOPBP1 may guide patient stratification for DDR-targeted therapies. These findings highlight TOPBP1 as both a functional regulator of DDR and a clinically relevant biomarker for optimizing combination treatments in PDAC [155].

Herbert et al. developed patient-derived PDAC cell line models with clinically relevant acquired resistance mechanisms through extended chemotherapy exposure. Transcriptomic and functional analyses revealed that resistance was associated with alterations in cell cycle checkpoint regulation, metabolic pathways, DDR, programmed cell death, and RS response. In these models, combination treatment with AZD6738 and olaparib produced synergistic anti-proliferative effects. Importantly, the sequence of administration influenced its efficacy: DDR-proficient models responded best when AZD6738 preceded olaparib, whereas DDR-deficient models were more sensitive to olaparib-first treatment. These findings provided in vitro evidence for a sequential ATR–PARP inhibitor strategy as a potential approach to overcome acquired chemoresistance in PDAC, offering a framework for future clinical investigation to mitigate dose-limiting toxicities associated with concurrent drug administration [156].

#### 5.1.2. Combination of ATR Inhibitors with Other Therapeutical Agents

High proliferative and protein synthesis rates in PDAC induce endoplasmic reticulum (ER) stress, with glucose-regulated protein 78 (GRP78) serving as a key regulator of the unfolded protein response (UPR) and contributing to tumor progression. Lee et al. evaluated the GRP78 inhibitor BOLD-100 and found that it induces PDAC cell death by activating the UPR pathway, leading to C/EBP homologous protein (CHOP)-dependent apoptosis. BOLD-100 also generates reactive oxygen species, promoting R-loop formation and triggering a DDR via the ATR–CHK1 signaling axis. Notably, BOLD-100 synergized with the ATR inhibitor AZD6738, enhancing anti-tumor efficacy compared to either agent alone in both in vitro and in vivo models. These findings indicated that targeting GRP78, particularly in combination with ATR inhibition, may offer a promising therapeutic approach for PDAC [157].

In conclusion, accumulating preclinical evidence underscores the therapeutic potential of targeting ATR in PDAC in rational combinations with DNA-damaging agents, DDR inhibitors, and novel chemotherapeutics. Across diverse experimental systems, including established cell lines, patient-derived organoids, xenografts, and genetically engineered mouse models, ATR inhibitors consistently disrupted cell-cycle checkpoint control, intensified RS, and impaired HR, thereby potentiating cytotoxicity of conventional agents such as gemcitabine, FOLFIRINOX, other fluoropyrimidines, and radiation. Importantly, synergy was also observed with PARP, WEE1, and GRP78 inhibition, while specific genetic contexts such as *ATM* deficiency or *TOPBP1* overexpression further refined therapeutic vulnerability. Collectively, these findings highlight ATR inhibition as a versatile strategy to overcome chemoresistance and enhance therapeutic efficacy in PDAC. A summary of representative preclinical studies is provided in Table 1.

### 5.2. CHK1 Inhibitors

Urrutia et al. combined inhibition of CHK1 using prexasertib with inhibition of the histone methyltransferase G9a (EHMT2) using BRD4770, aiming to disrupt replication fork stability through complementary mechanisms. Authors of the study assessed the effects of this combination in PDAC cell lines, patient-derived cells, 3D spheroids, and xenograft models. The combination demonstrated synergistic effects on replication-associated processes, including cell proliferation, DNA synthesis, S-phase progression, and DDR signaling, ultimately leading to enhanced cell death. Mechanistic analyses suggested that the synergy was mediated by the convergence of CHK1 and G9a functions at the ATR-RPA checkpoint pathway active during RS. These findings indicated that dual targeting of DNA damage checkpoints and epigenetic regulators may represent a promising strategy to induce replication catastrophe and control PDAC growth, highlighting a potential new therapeutic approach for this aggressive cancer [158].

Genetic screening approaches have been used to identify modulators of *KRAS*-mutant PDAC response to extracellular signal-regulated kinase (ERK) inhibition, revealing the role of the key components of the ATR–CHK1 DDR pathway. Pharmacological inhibition of CHK1 with prexasertib alone was shown to suppress growth and induce apoptosis in PDAC cell lines and organoids, an effect associated with reduced MYC expression. CHK1 inhibition also activated ERK and AMP-activated protein kinase (AMPK) signaling and increased autophagy, providing a mechanistic rationale for the enhanced efficacy of combining CHK1 inhibition with ERK inhibition and/or autophagy blockade using chloroquine. To further investigate the survival-promoting effects of CHK1 inhibition-induced ERK activation, a CRISPR-Cas9 loss-of-function screen targeting ERK substrates was performed. This screen identified replication timing regulatory factor 1 (RIF1), a component of NHEJ repair, as a critical mediator of the therapeutic effects. Suppression of RIF1 sensitized PDAC cells to apoptosis induced by CHK1 inhibition, while ERK inhibition reduced RIF1 expression and mirrored the effects of RIF1 depletion. Collectively, these findings indicated that dual suppression of DDR signaling enhances the therapeutic efficacy of ERK and/or autophagy inhibition in KRAS-mutant PDAC [159].

Strategies exploring combination therapies targeting CHK1 raised concerns about the potential cytotoxicity of the compounds towards normal cells. To identify alternative targets with similar functional impact but potentially greater specificity, a negative selection RNAi screen was conducted using small hairpin RNAs against over 10,000 genes in pancreatic cancer cell lines. This screen identified replication factor C (RFC)-related RAD17 (*RAD17*) as a gene whose suppression is synthetically lethal with gemcitabine. Subsequent functional studies demonstrated that *RAD17* knockdown significantly enhanced gemcitabine cytotoxicity in BxPC-3, MiaPaCa-2, and the primary JoPaca-1 cell line. Mechanistically, the combination of RAD17 inhibition and gemcitabine induced forced mitotic entry of S-phase-arrested cells, leading to asymmetric DNA segregation, DNA fragmentation, and cell death via mitotic catastrophe. Unlike CHK1 inhibition, *RAD17* knockdown alone did not cause abnormal DNA segregation, suggesting a more tumor-specific action. These findings proposed RAD17 as a promising target for gemcitabine-based combination therapies, potentially complementing or substituting CHK1 inhibition while limiting effects on normal cells [160].

### 5.3. ATM Inhibitors

Irreversible electroporation (IRE), a non-thermal ablative modality, has emerged as a treatment option for locally advanced and unresectable PDAC by applying high-intensity electric pulses to disrupt tumor cell membranes and induce cell death. However, heterogeneous electric field distribution within tumors can result in regions of reversible electroporation (RE), where tumor cells survive and contribute to relapse of the disease. Recent work demonstrated that RE, although non-lethal, induces DSBs and activates the DDR pathway. To exploit this vulnerability, a nanomedicine-based approach using reactive oxygen species-sensitive polymeric micelles co-loaded with the PARP inhibitor olaparib and the ATM inhibitor AZD0156 (M-TK-OA) was developed. This formulation simultaneously disrupted HR and NHEJ repair pathways, impairing clonogenic survival of pancreatic cancer cells following RE. In preclinical models, the combination of IRE with M-TK-OA significantly extended survival in both subcutaneous and orthotopic murine PDAC models and elicited CD8+ T cell-mediated antitumor immunity with durable memory responses. Mechanistic analyses further indicated that treatment efficacy was partly mediated through activation of the cyclic GMP-AMP synthase–stimulator of interferon genes (cGAS–STING) innate immune pathway. These findings highlight the therapeutic potential of combining IRE with dual PARP and ATM inhibition as a strategy to overcome tumor recurrence and improve outcomes in PDAC [161].

## 6. Clinical Studies

### 6.1. Completed Clinical Trials

In one of the first reports, Okazaki et al. sought to determine whether germline variations in DDR and checkpoint genes modulate therapeutic response and survival outcomes in patients treated with DNA-damaging agents. Given that gemcitabine-based chemoradiation induces RS and DNA strand breaks, the ATR–CHK1 and ATM–CHK2 signaling cascades play critical roles in mediating checkpoint activation, fork stabilization, and DNA repair, thereby influencing tumor cell fate under genotoxic stress. Functional single-nucleotide polymorphisms (SNPs) in these pathways may alter protein activity or expression, thereby modifying cellular capacity to repair DNA damage and influencing patient prognosis. To address this hypothesis, the authors analyzed six SNPs within *ATM*, *ATR*, *CHK1*, and *CHK2* in a cohort of 119 patients with potentially resectable pancreatic cancer who were treated on prospective clinical trials with neoadjuvant gemcitabine-radiation therapy, with or without gemcitabine-cisplatin induction. By correlating genotype with OS and treatment response, this study aimed to establish whether interpatient genetic variability within DDR signaling networks contributes to differential clinical outcomes, thus providing mechanistic insight into the role of ATR- and ATM-mediated checkpoint control in pancreatic cancer biology. The findings demonstrated that specific polymorphisms in *ATM* (G60A) and *CHK1* (G35A) were significantly associated with OS, whereas the *ATR* C340T variant showed borderline significance. Notably, patients harboring the *CHK1* 35AA genotype exhibited a twofold higher risk of death compared with *CHK1* 35GG/GA carriers, and a combined deleterious genotype effect revealed stepwise reductions in median OS from 31.0 to 16.2 to 10.5 months with increasing allele burden. These results provide evidence that inherited variation in DDR signaling genes modulates clinical response to gemcitabine-based therapy and survival in pancreatic cancer, underscoring the potential of ATR- and ATM-mediated pathways as prognostic markers and therapeutic targets [162].

The study by Li et al. evaluated 13 SNPs across eight DNA repair genes in a cohort of 92 patients with potentially resectable pancreatic adenocarcinoma treated with neoadjuvant concurrent gemcitabine and radiotherapy, with or without induction gemcitabine/cisplatin. Clinical endpoints included time to tumor progression and OS, which were analyzed in relation to genotype. The findings demonstrated that SNPs in ATP-dependent DNA helicase Q1 (*RecQ1*) (A159C), DNA repair and recombination protein RAD54-like (*RAD54L*) (C157T), *XRCC1* (R194W), and *ATM* (T77C) were significantly associated with OS, with log-rank *p* values of 0.001, 0.004, 0.001, and 0.02, respectively. A striking cumulative effect of these variants was observed: patients without any adverse alleles had a mean survival of 62.1 months, compared with 27.5, 14.4, and 9.9 months for those harboring one, two, or three or more at-risk alleles (*p* < 0.001). Gene–gene interaction analyses further highlighted RecQ1 as a modifier of other polymorphic effects, and multivariate models confirmed *RecQ1*, *RAD54L*, and *ATM*, though not *XRCC1*, as independent predictors of survival [163].

This study by Weekes et al. represented the first genotype-directed approach in pancreatic cancer, pre-selecting patients harboring the **TYMS2/2 polymorphism to receive capecitabine, based on prior evidence in colorectal cancer indicating increased efficacy and toxicity of 5-FU in this genotype. Of 82 screened patients, 16 possessed the TYMS*2/2 allele, but only 4 were enrolled due to the poor prognosis of second-line pancreatic cancer patients, competing clinical trials, and availability of capecitabine off-label. Despite the small sample size, treatment was associated with marked grade 2–3 non-hematologic toxicities, including diarrhea, nausea/vomiting, hand–foot syndrome, and mucositis, suggesting that factors beyond patient fitness or pharmacokinetic variability may contribute, potentially including additional polymorphisms affecting 5-FU metabolism or pharmacodynamics. Although no statistically significant associations were observed between *TYMS*, *ATM*, or *RecQ1* genotypes and OS, there was a trend toward poorer survival in metastatic patients harboring TYMS2/*2, consistent with previous observations in other malignancies. These findings highlight both the potential and the practical challenges of genotype-directed therapy in advanced pancreatic cancer, particularly in the second-line setting, and suggest that this strategy may be better suited for adjuvant or first-line metastatic therapy where patient enrollment and treatment feasibility are more favorable [164].

Personalized medicine approaches are particularly relevant in pancreatic cancer, where therapeutic options remain limited and outcomes are poor. The Individualized Molecular Pancreatic Cancer Therapy (IMPaCT) trial was designed to integrate genomic profiling into clinical management, leveraging sequencing data generated by the International Cancer Genome Consortium (ICGC). In its pilot phase, the trial evaluated the feasibility of acquiring suitable biospecimens and returning high-quality, actionable genomic data within a clinically relevant timeframe. Among 93 patients, tumor material was successfully collected from 76 cases, with 22 harboring potentially targetable alterations: 14 with *KRAS* wild-type tumors, 5 with human epidermal growth factor receptor 2 (*HER2*) amplification, 2 with *BRCA2* mutations, and 1 with an *ATM* mutation. The median turnaround time from patient consent to validated genomic results was 21.5 days. Exclusion from molecular analysis most commonly resulted from inadequate biopsy material or rapid clinical decline. These findings demonstrate the feasibility of real-time molecular stratification in pancreatic cancer and highlight key logistical challenges, including the importance of prescreening, maximizing patient recruitment, and expanding access to therapeutic trials based on actionable genomic findings [165].

So far one study (NCT03225105) investigated the ATM inhibitor in pancreatic cancer patients. In this phase I dose-escalation study, the orally administered selective ATM inhibitor M3541 was evaluated in combination with fractionated palliative radiotherapy in patients with solid tumors to determine the maximum-tolerated dose (MTD), recommended phase II dose (RP2D), safety, pharmacokinetics (PK), and preliminary antitumor activity. Fifteen patients received escalating doses of M3541 (50–300 mg) concurrent with radiotherapy (30 Gy in 10 fractions). The treatment was generally well tolerated, with only one patient experiencing dose-limiting toxicities that resolved with standard interventions. No grade ≥4 treatment-emergent adverse events (TEAEs) occurred, and all TEAEs were manageable without treatment discontinuation. Partial or complete responses were observed in three patients (20%). However, M3541 plasma levels did not show dose-proportional increases, and no clear relationship was identified between dose and pharmacodynamic markers, including phosphorylated ATM or immune cell counts. Due to the absence of a dose–response relationship and suboptimal PK profile, the MTD and RP2D were not established, and further clinical development of M3541 was discontinued [166].

Similarly, one study (NCT00839332) investigated the potential of CHK1 inhibitor in pancreatic cancer patients. Laquente et al. evaluated the combination of LY2603618 with gemcitabine versus gemcitabine alone in patients with unresectable stage II–IV pancreatic cancer. Ninety-nine patients were randomized 2:1 to receive either LY2603618 (230 mg) plus gemcitabine (1000 mg/m^2^) or gemcitabine alone. OS was the primary endpoint and was analyzed using both Bayesian augment control and traditional frequentist methods, alongside secondary endpoints including progression-free survival (PFS), overall response rate (ORR), duration of response, PK, and safety. Median OS was 7.8 months in the combination arm versus 8.3 months with gemcitabine alone, with a Bayesian posterior probability of superiority of 0.3, below the prespecified threshold of 0.8. No significant differences were observed in PFS, ORR, or duration of response. TEAEs, including nausea, thrombocytopenia, fatigue, and neutropenia, were comparable between the two groups, and PK targets for LY2603618 were achieved. These findings indicate that LY2603618 combined with gemcitabine does not improve clinical outcomes compared to gemcitabine monotherapy in unresectable pancreatic cancer [167].

### 6.2. Future and Ongoing Clinical Trials

ART0380 is a potent and selective oral ATR inhibitor under clinical investigation for patients with advanced or metastatic solid tumors, including pancreatic cancer. Phase I/IIa, open-label, multi-center trial (NCT04657068) has been designed to define the safety, tolerability, PK, and preliminary efficacy of ART0380, both as monotherapy and in combination with gemcitabine or irinotecan as agents that exacerbate RS and DNA damage. Importantly, this study also includes patients with *ATM*-deficient tumors and high-grade serous ovarian, primary peritoneal, or fallopian tube carcinomas, thereby addressing clinically relevant contexts of DDR dysfunction. By systematically evaluating ART0380 across these settings, the trial not only seeks to establish its recommended therapeutic dosing but also aims to expand the translational potential of ATR inhibition as a precision oncology approach. The outcomes of this study are expected to provide critical insights into the therapeutic window of ART0380 and its combinatorial value with standard chemotherapeutics, thereby advancing the clinical development of DDR-targeted therapies in difficult-to-treat malignancies.

AZD6738 is being investigated both as a single agent and in rational combinations with the PARP inhibitor olaparib and the immune checkpoint inhibitor durvalumab (antibody targeting programmed cell death protein 1/programmed death-ligand 1 (PD-1/PD-L1). Phase II trial (NCT03682289) was designed to evaluate the clinical activity of AZD6738 across biologically defined subgroups, including patients with AT-rich interactive domain-containing protein 1A (*ARID1A*) alterations or *ATM* deficiency in patients with renal cell carcinoma, urothelial carcinoma, all pancreatic cancers, endometrial cancer, and other solid tumors. By stratifying treatment arms according to immunohistochemistry and next-generation sequencing biomarkers, the study directly addresses the therapeutic vulnerabilities of DDR-deficient tumors while assessing the added benefit of combining ATR blockade with either synthetic lethality-inducing agents (olaparib) or immune checkpoint modulation (durvalumab). Primary endpoints include objective response rate and composite measures in metastatic castration-resistant prostate cancer, with secondary analyses of PFS, duration of response, and safety profiles across cohorts. This biomarker-driven approach has the potential to establish AZD6738 as both a monotherapy and a combinatorial backbone in precision oncology, while providing mechanistic insights into how DDR-targeted agents can synergize with PARP inhibition and immunotherapy.

Extrachromosomal DNA (ecDNA) is increasingly recognized as a driver of oncogene amplification, genomic instability, and therapeutic resistance in aggressive solid tumors. Targeting pathways that support ecDNA replication and survival represents a novel precision oncology strategy. BBI-355, an oral and selective CHK1 inhibitor, is being developed as an ecDNA-directed therapy (ecDTx) designed to disrupt RS responses in tumors harboring oncogene amplifications. In parallel, BBI-825, a potent oral inhibitor of ribonucleotide reductase (RNR), targets deoxynucleotide biosynthesis, further exacerbating RS and impairing DNA repair. First-in-human, phase I/II open-label trial (NCT05827614) was designed to establish the safety, PK, and RP2D of BBI-355 as monotherapy and in rational combinations with BBI-825 or other targeted agents, such as erlotinib and futibatinib. By enrolling patients with locally advanced or metastatic solid tumors that are refractory to standard therapy, including triple-negative breast cancer, high-grade serous ovarian and endometrial carcinomas, head and neck squamous cell carcinoma, small cell lung cancer, and other difficult-to-treat malignancies, including metastatic pancreatic cancer, the study directly addresses an area of high unmet clinical need. Beyond dose optimization, this trial has the potential to define ecDNA as a therapeutic vulnerability and establish BBI-355 as the first-in-class ecDTx agent, while also exploring synergistic strategies that couple RS induction with impaired nucleotide metabolism. The findings are anticipated to advance translational understanding of ecDNA-driven oncogenesis and pave the way for a new therapeutic paradigm in tumors with oncogene amplification.

In summary, emerging evidence underscores the therapeutic promise of targeting DDR vulnerabilities through inhibition of ATR and CHK1 signaling, either as monotherapy or in rational combinations with chemotherapeutics, PARP inhibition, or immune checkpoint blockade. Ongoing trials with ART0380, AZD6738, and BBI-355 (with or without BBI-825) exemplify this precision oncology approach, focusing on genomically defined subsets such as *ATM*-deficient tumors, *ARID1A*-altered cancers, and malignancies driven by oncogene amplification and extrachromosomal DNA. Collectively, these studies are designed to establish optimal dosing, define safety and tolerability, and evaluate preliminary efficacy across a spectrum of hard-to-treat solid tumors. A summary of these key ATR- and CHK1-directed clinical trials, including study design, rationale, and enrollment status, is presented in Table 2.

## 7. Conclusions and Prospects

Pancreatic cancer, which is overwhelmingly dominated (>90% cases) by pancreatic ductal adenocarcinoma (PDAC), exhibits pervasive RS and DDR liabilities that make the ATR–CHK1 axis an attractive therapeutic target. Although, the disease is dominated by oncogenic *KRAS* together with frequent *TP53*, cyclin-dependent kinase inhibitor 2A (*CDKN2A*), and SMAD family member 4 (*SMAD4*) alterations, a constellation that disrupts G1 control and enforces reliance on S/G2 surveillance and fork-stabilizing signals transmitted by ATR–CHK1 under RS, these represent a limited number of high-frequency driver events rather than a globally elevated mutational burden. In *KRAS*-mutant PDAC models, CHK1 safeguards genome integrity and its inhibition precipitates replication fork collapse and mitotic catastrophe even in *TP53*-defective settings, underscoring a *KRAS*-driven dependency on ATR–CHK1 for survival during RS. Concurrent MYC activity, reported in subsets of PDAC and linked to S-phase transit, further amplifies RS and ATR–CHK1 signaling, providing an additional mechanistic rationale for checkpoint blockade in this setting [168,169,170,171,172].

PDAC is characterized by low immunogenicity, limited tumor mutation burden (TMB), scarce neoantigen expression and immunosuppressive tumor microenvironment (rich in CD4+ FOXP3+ regulatory T-cells (TREGs), M2-like tumor-associated macrophages (TAMs), myeloid-derived suppressor cells (MDSCs), and fibroblast activation protein-positive cancer-associated fibroblasts), which collectively constrain the efficacy of immunotherapies such as immune checkpoint blockade. Conventional approaches to elevate TMB through DNA-damaging agents are restricted by intact DNA repair mechanisms, leading to incomplete destabilization of the genome and insufficient neoantigen generation [173,174,175]. In contrast, tumors with germline *BRCA1/2* or other HRD display heightened sensitivity to platinum-based chemotherapy, reflecting their impaired capacity to repair DSBs. Beyond canonical HR genes, mutations in broader DDR genes also contribute to HRD, but a lack of standardized methods for defining HRD status and the absence of a consensus HR-DDR gene panel have hindered clinical translation. To address this gap, a novel genomic classification framework integrating whole-exome and whole-genome sequencing was developed to stratify PDAC into HRD-positive and HRD-negative subgroups. In a cohort of 89 PDACs, HRD-positive tumors harbored significantly more HR-DDR variants, particularly in *RAD51B*, *BRCA2*, and *ATM*, compared to HRD-negative tumors. Importantly, HRD-positive PDACs exhibited elevated TMB and neoantigen load, features typically associated with increased immunogenicity. However, paradoxically, these tumors demonstrated reduced CD8+ T cell infiltration and diminished T cell receptor (TCR) clonal diversity, suggesting an immune-exclusion phenotype that may explain the limited efficacy of immune checkpoint inhibitors in this setting despite higher TMB. This work is novel in establishing a robust genomic approach to define HRD status in PDAC and in linking HRD-associated mutational landscapes to immune microenvironmental features. Clinically, these findings underscore the need for combination strategies, such as DDR inhibitors, STING agonists, or T cell-engaging therapies, to overcome barriers to effector T cell infiltration and clonal expansion in HRD-positive PDAC, thereby unlocking the therapeutic potential of ICIs in this molecularly defined subgroup [176].

Therefore, therapeutic targeting of DDR kinases offers a promising strategy to overcome the immune-refractory phenotype of PDAC. Inhibition of the ATR–CHK1 axis have been shown to disrupt RS tolerance, precipitating premature mitotic entry, micronuclei formation, and cytosolic dsDNA accumulation that activates the cGAS–STING pathway and downstream type I interferon (IFN-I) signaling in different types of cancer [177,178]. This cascade promotes dendritic cell activation, effector T-cell recruitment, and a favorable effector/Treg balance within the typically desmoplastic, “immune-cold” PDAC microenvironment, while also sensitizing tumors to radiotherapy by amplifying IFN-I and proinflammatory chemokine production (e.g., CCL2, CCL3, CCL5, CXCL10; for full names see abbreviations section) [177]. In *TP53*-mutant PDAC models, the selective ATR inhibitor VE-821 demonstrated variable efficacy, with epithelial BxPC-3 cells exhibiting greater sensitivity compared to mesenchymal PANC-1 cells, which displayed heightened migratory capacity and elevated PD-L1 and CD44 expression. ATR blockade in PANC-1 cells further induced epithelial-to-mesenchymal transition (EMT), accompanied by upregulation of PD-L1 and CD44, whereas BxPC-3 cells did not undergo these adaptive changes. Importantly, genetic or pharmacologic attenuation of PD-L1 suppressed ATR inhibitor–induced EMT, impaired migration, downregulated CD44, and enhanced DNA damage, thereby restoring sensitivity to VE-821. Mechanistically, PD-L1 inhibition partially reversed AKT/ERK pathway activation, linking checkpoint signaling to both EMT and DDR resilience. Transcriptomic analyses further revealed a positive correlation between PD-L1 expression and EMT transcriptional programs in patient-derived datasets, reinforcing the clinical relevance of this axis. These findings establish a novel role for PD-L1 in modulating the therapeutic response to ATR inhibition in mesenchymal PDAC, and suggest that concurrent blockade of PD-L1 may not only augment the cytotoxic efficacy of ATR inhibitors but also mitigate pro-metastatic EMT programs, thereby broadening the translational potential of DDR-targeted immuno-oncology strategies [179].

Complementarily, ATM–CHK2 inhibition elicits innate immune priming through a distinct cGAS–STING-independent mechanism, mediated instead by TANK-binding kinase 1 (TBK1) and SRC proto-oncogene, non-receptor tyrosine kinase (SRC), and synergizes with radiation to further potentiate IFN-I signaling, PD-L1 upregulation, and CD8+ T-cell infiltration in pancreatic cancer [180,181]. The upregulation of PD-L1 in cancer cells may increase the number of epitopes available for anti-PD-L1 agents to bind, which could further contribute to the therapeutic efficacy of combination treatments of DDR inhibitors with immune checkpoint inhibitors. Consistently, tumors with high PD-L1 expression often respond favorably to anti-PD-1/PD-L1 therapies [182,183,184]. Importantly, WEE1–ATM co-inhibition also dampens PD-L1 and CKLF-like MARVEL transmembrane domain-containing protein 6 (CMTM6) expression, counteracting adaptive immune resistance mechanisms, while IFN-driven replication stress primes PDAC cells for lethal ATR inhibition, establishing a context-dependent vulnerability [184,185,186].

Collectively, these findings highlight that pharmacologic blockade of ATR–CHK1 or ATM–CHK2 pathways, particularly in combination with radiotherapy and immune checkpoint blockade, can reprogram PDAC’s immunologic milieu by coupling DNA damage-induced cytotoxicity with innate immune activation, providing a mechanistic rationale for integrating DDR inhibitors into immuno-oncology strategies for this otherwise treatment-refractory malignancy.

In line with these findings, a novel nanomedicine platform (MOFDOX&siATR) has been developed that integrates a metal–organic framework (MOF) delivery system with dual functionality: encapsulation of the genotoxic chemotherapeutic doxorubicin to induce DNA mutations, and co-delivery of ATR-targeting siRNA to inhibit DNA repair. This dual-pronged strategy not only enhances DNA instability but also sustains the accumulation of mutations, effectively amplifying tumor antigenicity. Importantly, MOFDOX&siATR reprograms the tumor gene expression landscape and induces the generation of novel neoantigens, including ATPase phospholipid transporting 8B1 (ATP8B1)-derived epitopes, thereby boosting the immunogenic potential of PDAC cells. The conceptual innovation lies in leveraging nanotechnology to couple mutagenesis with concurrent blockade of DDR pathways, a previously unexplored approach to systematically increase neoantigen presentation in “cold” tumors. Clinically, such a platform holds promise for converting PDAC into a more immunoresponsive malignancy, creating a therapeutic window to synergize with checkpoint inhibitors and other immunotherapies. This study thus introduces a first-in-class DNA stability-intervening nanoregulator that may overcome one of the fundamental limitations in PDAC immunotherapy, paving the way for translational development of combination regimens aimed at durable immune control [173].

Although preclinical studies in PDAC have consistently demonstrated potent antitumor activity of DDR inhibitors, including agents targeting ATR, CHK1, WEE1, PARP, and ATM, their translation into clinical practice has been constrained by a combination of biological, pharmacological, and safety-related challenges. The DDR functions as an essential tumor-suppressive barrier during early tumorigenesis, where oncogene-driven replication stress and endogenous DNA damage activate checkpoint signaling to enforce senescence or apoptosis. However, selective attenuation or rewiring of DDR pathways during tumor evolution paradoxically enables the survival of genomically unstable cells, fostering malignant progression and therapeutic resistance. This dependency is further accentuated in cancer stem cell populations, which frequently exhibit constitutively elevated DDR activity and enhanced checkpoint control, conferring resistance to DNA-damaging therapies and promoting disease relapse. Therefore, the fundamental limitation shared across DDR-targeted therapies is the restricted therapeutic index, as these pathways are indispensable for genome maintenance not only in cancer cells but also in normal proliferating tissues. Consequently, dose-limiting toxicities, most notably hematologic adverse events such as anemia, thrombocytopenia, and neutropenia, as well as gastrointestinal toxicities including nausea, vomiting, and diarrhea, have been frequently observed in early-phase clinical trials, particularly when DDR inhibitors are combined with cytotoxic chemotherapy or radiation. In rare but clinically significant cases, prolonged DDR inhibition has been associated with secondary myelodysplastic syndromes (MDS) and acute myeloid leukemia (AML), especially in heavily pretreated patients. Beyond toxicity, functional redundancy and plasticity within DDR networks often attenuate therapeutic efficacy, enabling tumor cells to bypass single-node inhibition through compensatory activation of parallel pathways such as ATM–CHK2 signaling, WEE1-mediated checkpoint control, or enhanced replication fork protection. Tumor heterogeneity further complicates clinical translation, as only subsets of PDAC exhibit actionable vulnerabilities such as complete ATM loss, profound replication stress, or homologous recombination deficiency, thereby limiting the generalizability of preclinical findings [187,188,189].

Emerging evidence indicates that ATM dependency can arise independently of classical homologous recombination deficiency, particularly in the context of oncogenic and metabolic stress induced by loss of key tumor suppressors such as PTEN. PTEN-deficient cells display elevated endogenous DNA damage and constitutive ATM activation, reflecting a heightened reliance on ATM signaling to maintain genome integrity. Although PTEN loss has been associated with impaired homologous recombination in some settings, RAD51 expression and function remain intact in several PTEN-deficient models, suggesting that increased genomic instability arises through alternative mechanisms. Notably, PTEN deficiency is closely linked to enhanced oxidative stress, which drives DNA base oxidation and accumulation of double-strand breaks, thereby creating a cellular state in which ATM activity becomes essential for survival. Consistent with this model, antioxidant treatment partially rescues the cytotoxic effects of ATM inhibition in PTEN-deficient cells, underscoring oxidative DNA damage as a critical mediator of synthetic lethality and expanding the therapeutic rationale for ATM inhibition beyond canonical DNA repair-deficient contexts [190].

Importantly, the lack of validated predictive and pharmacodynamic biomarkers has hindered optimal patient stratification, dose selection, and scheduling, contributing to variable clinical outcomes despite robust preclinical efficacy. Interpatient variability in toxicity risk, potentially influenced by factors such as clonal hematopoiesis or underlying DDR alterations, further underscores the need for biomarker-guided treatment strategies. Collectively, these considerations explain why promising preclinical results have not uniformly advanced to late-phase clinical testing and highlight that the successful clinical implementation of DDR inhibitors in PDAC will require careful balancing of efficacy and safety through rational combination regimens, optimized dosing schedules, and biomarker-driven patient selection. This topic was discussed by other authors [187,188,189].

**Figure 4:**
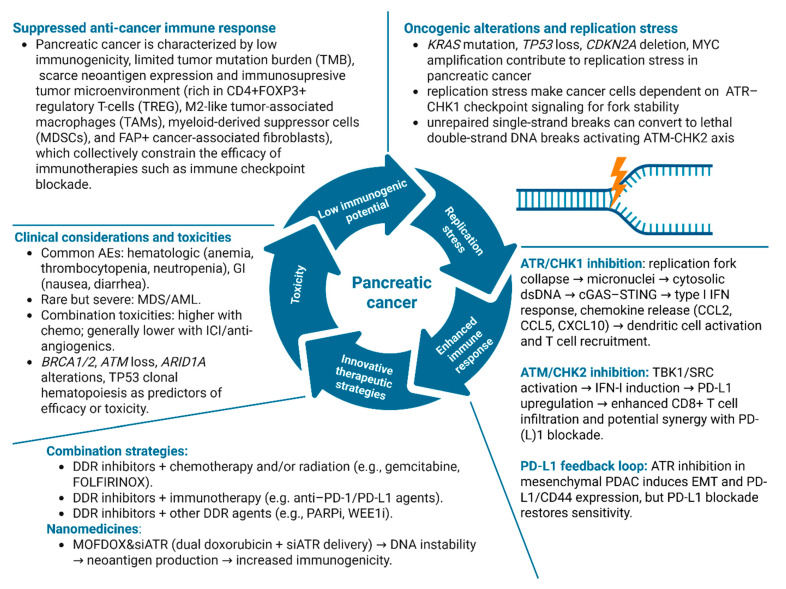
Therapeutic rationale for targeting DNA damage response pathways in pancreatic cancer. Pancreatic cancer exhibits pervasive replication stress driven by oncogenic KRAS (Kirsten rat sarcoma viral oncogene homolog), TP53 (tumor protein p53) loss, and MYC (v-myc avian myelocytomatosis viral oncogene homolog) activity, rendering tumor cells dependent on ataxia telangiectasia and Rad3-related (ATR)–checkpoint kinase 1 (CHK1) and ataxia telangiectasia mutated (ATM)–checkpoint kinase 2 (CHK2) checkpoint signaling for survival. Pharmacologic inhibition of ATR–CHK1 induces replication fork collapse and micronuclei formation, activating cyclic GMP-AMP synthase–stimulator of interferon genes (cGAS–STING) and type I interferon (IFN-I) responses; ATM–CHK2 inhibition engages TANK-binding kinase 1 (TBK1)/SRC proto-oncogene, non-receptor tyrosine kinase (SRC)-dependent IFN-I signaling, enhancing programmed death-ligand 1 (PD-L1) expression and CD8+ T cell infiltration. Despite high tumor mutation burden (TMB) in homologous recombination-deficient (HRD)-positive cancer, immune exclusion phenotypes with low T cell infiltration limit immune checkpoint blockade efficacy. Rational combinations include DNA damage response inhibitors with chemotherapy, poly (ADP-ribose) polymerase (PARP) or WEE1 G2 checkpoint kinase (WEE1) inhibition, immune checkpoint blockade, or innovative nanomedicine platforms (e.g., metal–organic framework doxorubicin and small interfering RNA targeting ATR (MOFDOX&siATR)) that enhance DNA instability and neoantigen generation. Clinical translation requires balancing efficacy with toxicity management; hematologic and gastrointestinal adverse events predominate, with rare but severe risks such as therapy-related myelodysplastic syndrome/acute myeloid leukemia (MDS/AML), emphasizing the need for biomarker-driven patient selection. Created in BioRender. Kciuk, M. (2025) https://BioRender.com/m8pxue9 (accessed on 30 December 2025).

In conclusion, accumulating preclinical and early clinical evidence focused mainly on ATR inhibition as a versatile and clinically actionable strategy to exploit DDR vulnerabilities in PDAC and other solid tumors. Across diverse preclinical models, ATR blockade disrupted replication stress tolerance, impaired HR, and disabled checkpoint control, thereby enhancing cytotoxicity from DNA-damaging agents such as gemcitabine, FOLFIRINOX, fluoropyrimidines, and radiotherapy. Synergistic activity has also been demonstrated in combination with other DDR-targeting agents, including PARP and WEE1 inhibitors, and with emerging targets such as GRP78. Importantly, specific genomic contexts, such as *ATM* deficiency, *ARID1A* alterations, or oncogene-driven replication stress, further refine therapeutic sensitivity, providing a rationale for patient stratification. Early-phase clinical trials with ATR inhibitors, including ART0380, AZD6738, and BBI-355 (alone or in combination with BBI-825) are beginning to establish safety, tolerability, and preliminary efficacy in genomically defined subsets of refractory cancers. Together, these findings position ATR inhibition as a promising precision oncology strategy capable of overcoming chemoresistance, augmenting the efficacy of standard therapies, and potentially enabling novel combinatorial regimens. Future studies should prioritize biomarker-driven trial designs, integration with immunotherapy, and careful management of toxicity to fully realize the therapeutic potential of ATR-CHK1 and ATM-CHK2 targeted approaches in pancreatic cancer (Figure 4).

## Figures and Tables

**Table 1:** Preclinical studies examining the use of ATR inhibitors for pancreatic cancer treatment.

Combination	Rationale	System/Models	Key Findings	Source
VE-822 + radiation/gemcitabine	Enhancement of the therapeutic efficacy of chemo- and radiotherapy by blocking ATR signaling	In vitro and in vivo xenografts: pancreatic cancer (MiaPaCa-2 and PSN-1) and primary PDAC line (PancM) + normal human fibroblast cells (MRC5: CCL171; and HFL-1: CCL153)	Disrupted checkpoint maintenance; ↑ DNA damage; inhibited HR; ↓ clonogenic survival; tumor growth delay without added normal tissue toxicity	[141]
VE-822 + FOLFIRINOX	Overcoming resistance to the chemotherapeutic regimen to improve its efficacy through ATR inhibition	Primary pancreatic cancer cell line (TC25) obtained from an untreated PDAC. The Pancpec, P4604 and P7054 cell lines were derived from PDX of human primary pancreatic tumour, peritoneum and liver metastasis specimens. The mouse KPC-Luc cell line and the immortalised CAF3 cell line. CAFs isolated from human PDAC samples using the outgrowth method.	Strong synergy between drugs observed independent of *BRCA1*/*2*/*ATM* status; effective in resistant models; ↑ apoptosis; remodeling of TME (↓ CAF, ↑ immune infiltration)	[142]
AZ20 + gemcitabine	ATR inhibition as a means to amplify gemcitabine-induced DNA damage in cancer cells	In vitro: AsPC-1, BxPC-3, CFPAC-1, HPAC and MIAPaCa-2 human pancreatic cancer cell lines	AZ20 reduced CHK1 phosphorylation; caused S- and G2/M arrest, and exhibited synergy with gemcitabine, with promotion of cell death. RRM2 upregulation was blocked through the use of the combination	[143]
AZD6738 + gemcitabine	ATR inhibition as a means to amplify gemcitabine efficacy and overcome resistance	In vitro and in vivo xenografts/KPC model: human pancreatic cancer cells (MIA PaCa-2, Panc-1, SW1990, Capan-1, AsPC-1, HPAF-II, Capan-2), murine pancreatic cancer cells (K8484, DT8082, TB31456, TB32048 previously established from KRasG12D; p53R172H; Pdx1-Cre mice), KPCFT79653 established from a KrasG12D; Trp53R270H; Brca2Tr/Δ11; Pdx-Cre (KPCB) mouse	AZD6738 prevented gemcitabine-induced CHK1 activation and RRM2 accumulation. Synergistic anticancer activity with a near-complete loss of clonogenicity, significant tumor regression, and extended survival in xenograft models	[144]
AZD6738 + gemcitabine (*ATM* loss context)	Study to assess contribution of *ATM* deficiency to replication stress	In vitro and in vivo xenografts/KPC model: human pancreatic cell lines: AsPC-1, MIA PaCa-2, PANC-1 and HPAF-II, murine pancreatic cancer cells: K8484 and DT8082 established from KPC mice of 129/SvJae/C57Bl/6 background	Only complete abrogation of ATM function, achieved by either pharmacological blockade or CRISPR-mediated knockout, but not by partial reduction with siRNA, conferred marked sensitivity to the ATR inhibitor–gemcitabine combination. In the absence of ATM, this treatment intensified replication catastrophe, while phosphorylation of CHK2 (T68) and KAP1 (S824) remained sustained through DNA-PK signaling. In vivo, the ATRi/gemcitabine regimen produced only growth delay in xenografts with intact ATM, whereas ATM-null tumors underwent regression in NSG mice.	[145]
Elimusertib + gemcitabine	Identification of synergistic compounds through large-scale screening	Pancreatic cancer cell lines: MiaPaCa-2, AsPC-1, PSN-1, BxPC-3, PANC-1, FAMPAC, Capan-1, PaTu-8988-T, Dan-G, HuP-T4 Suit2-07, Colo-357, HPDE and patient-derived organoids	Strong synergy between elimusertib and gemcitabine in the majority of cell lines, evidenced by blocked CHK1 phosphorylation	[146]
VE-822 or olaparib in *ATM*-deficient PDAC	Exploitation of synthetic lethality between ATR, PARP-1, and ATM	In vitro and in vivo: heterotopic and orthotopic mouse models	ATM loss confers mitotic defects and genomic instability, making *ATM*-deficient PDAC sensitive to PARPi (olaparib) and ATRi (VE-822) combination	[147]
AZD6738 + S-1	Targeting ATR as a means of replication stress enhancement in PDAC	In vitro: pancreatic cell lines (BxPC-3, SUIT-2, PANC-1, MiaPaCa-2) and xenograft models	Synergistic PDAC cell proliferation suppression, increase in cell apoptosis, blocked ATR–CHK1 signaling with xenograft growth markedly reduced	[148]
CF10 ± ATR (AZD6738)/WEE1 (AZD1775) inhibitors	Synergistic use of potent next-generation fluoropyrimidine agent and targeted therapies suppressing ATR and WEE1 activities to augment replication stress	In vitro: pancreatic cancer cell lines: BXPC3, Capan-1/2, HPAF-II, HS 766T, AsPC-1†, MIA PaCa-2†, Panc-1†, 4853-T, 7171-T and primary PDAC patient-derived models	CF10 exhibited ~308× more potent activity than 5-FU. Efficacy was potentiated by ATR/WEE1 inhibitors and reversed by dNTPs supports RS mechanism involved in the cellular response to combinations	[149]
WEE1 (MK-1775), ATR (VE-821), CHK1 (SB 218078) inhibitors + gemcitabine	Exploit checkpoint inhibition to enhance cytotoxicity of gemcitabinebine	In vitro: Panc1 (human pancreatic epithelioid carcinoma) and U2OS (human osteosarcoma)	DDR inhibitors enhanced gemcitabine activity. WEE1 inhibition exhibited the strongest potentiating potential; WEE1 inhibition required CDK1/2 and PLK1 to reduce ATR/CHK1 axis pathway activity. Furthermore, reduction in claspin and CtIP was observed.	[150]
Olaparib + AZD6738 + radiation	Extending PARPi and radiation efficacy to HR-proficient PDAC through ATR inhibition	In vitro: HR proficient (MiaPaCa2, Panc1) and HR deficient (Capan1) pancreatic cell lines. CRISPR-Cas9 PARP1 KO cell lines. Pancreatic cancer cell xenografts	Synergy was observed independent of HR status. PARP1–DNA complexes are central to radiosensitization. Increase in DSBs prevalence, RS and significant tumor growth delay was observed in HR-proficient models following treatment with the drug combination.	[154]
Olaparib + AZD6738 in high TOPBP1 PDAC	TOPBP1 as predictive biomarker for augmented combinatorial efficacy of olaparib and AZD6738 in PDAC	In vitro: pancreatic cells (Patu8988, BXPC3, AsPC-1, PANC-1, CFPAC, and MIAPaCa-2), primary cell lines (0001, 0037, 0049 derived from PDAC patients, xenografts	High TOPBP1 correlated with poor prognosis. Combination treatment induced TP53-dependent apoptosis and was most effective in TOPBP1-high tumors	[155]
AZD6738 + Olaparib (sequential dosing)	Combination treatment to overcome chemoresistance	Patient derived cell lines and BRCA2 revertant Capan1 cell lines	Drugs exhibited synergy. Sequence-dependent efficacy was noted (ATRi followed by PARPi showed most profound activity in DDR-proficient cancers) which supports sequential over concurrent use	[156]
AZD6738 + BOLD-100 (GRP78 inhibitor)	Exploiting ER stress-induced DDR to boost treatment efficacy	In vitro and in vivo: pancreatic cancer cell lines (SNU-213, SNU-324, SNU-2918, PANC-1, Capan-1, Capan-2, AsPC-1, and MIA PaCa2,HPAF-II cell)	BOLD-100 induced CHOP-dependent apoptosis and ROS accumulation which triggered ATR–CHK1 axis activation and synergized with AZD6738	[157]

Abbreviations: 5-FU—5-fluorouracil; ATM—ataxia-telangiectasia mutated; ATR—ataxia-telangiectasia and Rad3-related; ATRi—ATR inhibitor; BRCA1/2—breast cancer type 1/2 susceptibility protein; CAF—cancer-associated fibroblast; CDK1/2—cyclin-dependent kinase 1/2; CHK1—checkpoint kinase 1; CHK2—checkpoint kinase 2; CRISPR—clustered regularly interspaced short palindromic repeats; CtIP—C-terminal binding protein (CtBP) interacting protein; dNTP—deoxynucleotide triphosphate; DDR—DNA damage response; DNA-PK—DNA-dependent protein kinase; DSB—double-strand break; ER—endoplasmic reticulum; FOLFIRINOX—folinic acid (leucovorin), fluorouracil, irinotecan, and oxaliplatin; GRP78—glucose-regulated protein 78; HR—homologous recombination; KAP1—KRAB-associated protein 1; KO—knockout; KPC—genetically engineered mouse model with Kras^G12D, Trp53^R172H, and pancreas-specific Cre recombinase; NSG—NOD scid gamma; PARP—poly (ADP-ribose) polymerase; PARPi—PARP inhibitor; PDAC—pancreatic ductal adenocarcinoma; PDX—patient-derived xenograft; PLK1—Polo-like kinase 1; RAD51—DNA repair protein RAD51 homolog; RRM2—ribonucleotide reductase M2 subunit; ROS—reactive oxygen species; S-1—tegafur, gimeracil (CDHP), and oteracil potassium (Oxo); siRNA—small interfering RNA; TME—tumor microenvironment; TOPBP1—DNA topoisomerase II binding protein 1; TP53—tumor protein p53; UPR—unfolded protein response; VE-821, VE-822, AZ20, AZD6738, Elimusertib—small-molecule ATR inhibitors; WEE1—G2 checkpoint kinase; ↑—increase(d); ↓—decrease(d).

**Table 2:** Ongoing clinical trials investigating ATR-CHK1 inhibitors in pancreatic cancer treatment.

Agent/Trial Title	NCT Number	Description & Rationale	Status	Estimated Enrollment
ART0380—A Phase I/IIa, Open-label, Multi-center Study to Assess the Safety, Tolerability, Pharmacokinetics and Preliminary Efficacy of the ATR Kinase Inhibitor ART0380 Administered Orally as Monotherapy and in Combination to Patients With Advanced or Metastatic Solid Tumors	NCT04657068	Potent, selective oral ATR inhibitor under investigation in advanced/metastatic solid tumors, including pancreatic cancer. Trial evaluates ART0380 as monotherapy and in combination with gemcitabine or irinotecan to exacerbate replication stress/DNA damage. Includes patients with *ATM*-deficient tumors and high-grade serous ovarian, primary peritoneal, or fallopian tube carcinomas. Aims to establish recommended dosing, assess safety/tolerability, and expand the translational role of ATR inhibition.	Recruiting	597
AZD6738 (Ceralasertib)—Phase II Trial of Ceralasertib (AZD6738) Alone and in Combination With Olaparib or Durvalumab in Patients With Selected Solid Tumor Malignancies	NCT03682289	Oral ATR inhibitor studied as monotherapy and in combination with PARP inhibitor (olaparib) or programmed cell death protein 1/programmed death-ligand 1 (PD-1/PD-L1) immune checkpoint inhibitor (durvalumab). Focuses on biomarker-defined subgroups (*ARID1A*-altered, *ATM*-deficient tumors), including RCC, urothelial carcinoma, pancreatic, endometrial, and other solid tumors. Primary endpoints include ORR and composite measures; secondary endpoints include PFS, DOR, and safety. Designed to establish ATR inhibition as monotherapy and as a combinatorial backbone in DDR-deficient cancers.	Recruiting	89
BBI-355 (± BBI-825)—Study of the CHK1 Inhibitor BBI-355, an ecDNA-directed Therapy (ecDTx), and the RNR Inhibitor BBI-825, in Subjects With Tumors With Oncogene Amplifications (POTENTIATE)	NCT05827614	First-in-class ecDNA-directed therapy (CHK1 inhibitor BBI-355) investigated alone or with RNR inhibitor BBI-825 and other agents (e.g., erlotinib, futibatinib). Designed to disrupt replication stress response and DNA repair in ecDNA-driven oncogene-amplified tumors. Enrolling patients with advanced/metastatic refractory solid tumors, including TNBC, high-grade serous ovarian/endometrial carcinoma, HNSCC, SCLC, sarcomas, and metastatic pancreatic cancer. Trial aims to define safety, optimal dosing, and translational value of ecDNA targeting as a novel therapeutic paradigm.	Recruiting	127

Abbreviations: ATR—Ataxia Telangiectasia and Rad3-related; ATM—Ataxia Telangiectasia Mutated; CHK1—Checkpoint Kinase 1; DDR—DNA Damage Response; ecDNA—Extrachromosomal DNA; ecDTx—Extrachromosomal DNA-directed Therapy; HNSCC—Head and Neck Squamous Cell Carcinoma; ORR—Objective Response Rate; PFS—Progression-Free Survival; DOR—Duration of Response; PARP—Poly (ADP-ribose) Polymerase; RCC—Renal Cell Carcinoma; RNR—Ribonucleotide Reductase; SCLC—Small Cell Lung Cancer; TNBC—Triple-Negative Breast Cancer.

## Data Availability

No new data were created or analyzed in this study. Data sharing is not applicable to this article.

## Abbreviations

The following abbreviations are used in this manuscript:

53BP1	TP53-binding protein 1
5-FU	5-fluorouracil
9-1-1	RAD9-RAD1-HUS1 complex
AML	acute myeloid leukemia
AMPK	AMP-activated protein kinase
ARID1A	AT-rich interactive domain-containing protein 1A
ASIR	age-standardized incidence rate
ASMR	age-standardized mortality rate
ATM	ataxia-telangiectasia mutated kinase
ATP8B1	ATPase phospholipid transporting 8B1
ATR	ataxia telangiectasia and rad3-related
ATRIP	ATR-interacting protein
BAX	BCL2 associated X, apoptosis regulator
BCL-2	B-cell lymphoma 2
BCL-XL	B-cell lymphoma-extra large
BMI	body mass index
BRCA1/2	breast cancer type 1/2 susceptibility protein
CAFs	cancer-associated fibroblasts
CBP/p300	CREB-binding protein (CBP) and its paralog p300
CCL2	C–C motif chemokine ligand 2
CCL3	C–C motif chemokine ligand 3
CCL5	C–C motif chemokine ligand 5
CD44	Cluster of Differentiation 44
CDC25	cell division control 25 phosphatases
CDK	cyclin-dependent kinases
CDKN2A	cyclin-dependent kinase inhibitor 2A
cGAS–STING	GMP-AMP synthase–stimulator of interferon genes
CHK1	checkpoint kinase 1
CHK2	checkpoint kinase 2
CHOP	C/EBP homologous protein
CMTM6	CKLF-like MARVEL transmembrane domain-containing protein 6
CRISPR	clustered regularly interspaced short palindromic repeats
CRISPR-Cas9	Clustered Regularly Interspaced Short Palindromic Repeats–CRISPR-associated protein 9
CtBP	C-terminal binding protein
CtIP	CtBP interacting protein
CXCL10	C–X–C motif chemokine ligand 10
DALYs	disability-adjusted life years
DDR	DNA damage response
DNA-PK	DNA-dependent protein kinase
DNA-PKcs	DNA-dependent protein kinase catalytic subunit
dNTP	deoxynucleotide triphosphate
DOR	duration of response
DSBR	double-strand DNA break repair
DSBs	DNA double-strand breaks
EcDNA	extrachromosomal DNA
ecDTx	ecDNA-directed therapy
EHMT2	histone methyltransferase G9a
EMT	epithelial-to-mesenchymal transition
ER	endoplasmic reticulum
ERK	extracellular signal-regulated kinase
ETAA1	Ewing tumor-associated antigen 1
FOL	folinic acid
GRP78	glucose-regulated protein 78
HER2	human epidermal growth factor receptor 2
HNSCC	head and neck squamous cell carcinoma
HR	homologous recombination
HRD	homologous recombination deficiency
ICGC	International Cancer Genome Consortium
IFN-1	type I interferon
IHC	Immunohistochemistry
IMPaCT	The Individualized Molecular Pancreatic Cancer Therapy
IRE	irreversible electroporation
IRI	irinotecan
KAP1	KRAB-associated protein 1
KO	knockout
KPC	genetically engineered mouse model with Kras^G12D, Trp53^R172H, and pancreas-specific Cre recombinase
LIG4	ligase IV
LIGIII	DNA ligase III
MDC-1	mediator of DNA damage checkpoint protein 1
mDDRi	multi-DDR interference
MDM2	mouse double minute 2 homolog
MDS	myelodysplastic syndromes
MDSCs	myeloid-derived suppressor cells
MOF	metal–organic framework
MRN	MRE11-RAD50-NBS1 complex
MTD	maximum-tolerated dose
nal-IRI	liposomal irinotecan
NBS1	Nibrin
NGS	next-generation sequencing
NHEJ	non-homologous end joining
OS	overall survival
OXA	oxaliplatin
P21	cyclin-dependent kinase inhibitor 1A
PALB2	partner and localizer of BRCA2
PAR	poly(ADP-ribose)
PARP	poly (ADP-ribose) polymerase
PARPi	PARP inhibitor
PCNA	proliferating cell nuclear antigen
PD-1/PD-L1	programmed cell death protein 1/programmed death-ligand 1
PDAC	pancreatic ductal adenocarcinoma
PDX	patient-derived xenograft
PFS	progression-free survival
PK	Pharmacokinetics
PLK1	Polo-like kinase 1
PNKP	polynucleotide kinase/phosphatase
POLB	DNA polymerase β
pRB	retinoblastoma protein
PUMA	p53 up-regulated modulator of apoptosis
RAD17	replication factor C (RFC)-related RAD17
RAD51	DNA repair protein RAD51 homolog/RAD51 homolog 1
RAD54L	DNA repair and recombination protein RAD54-like
RCC	renal cell carcinoma
RE	reversible electroporation
RecQ1	ATP-dependent DNA helicase Q1
RIF1	replication timing regulatory factor 1
RNF4	ring finger protein 4
RNF8	ring finger protein 8
RNF168	ring finger protein 168
RNR	ribonucleotide reductase
ROS	reactive oxygen species
RP2D	recommended phase II dose
RPA	replication protein A
RRM2	ribonucleotide reductase M2 subunit
RS	replication stress
S-1	tegafur, gimeracil (CDHP), and oteracil potassium (Oxo)
SCLC	small cell lung cancer
siRNA	small interfering RNA
SMAD4	SMAD family member 4
SNPs	single-nucleotide polymorphisms
SRC	SRC proto-oncogene, non-receptor tyrosine kinase
SSBR	single-strand DNA break repair
SSBs	single-strand breaks
ssDNA	single-stranded DNA
SWI/SNF	SWItch/Sucrose Non-Fermentable
TAMs	M2-like tumor-associated macrophages
TBK1	TANK-binding kinase 1
TCR	T cell receptor
TEAEs	treatment-emergent adverse events
TIP60	Tat-interactive protein 60 kDa
TMB	tumor mutation burden
TME	tumor microenvironment
TNBC	triple-negative breast cancer
TOPBP1	DNA topoisomerase 2-binding protein 1
TP53	cellular tumor antigen P53
TREGs	regulatory T-cells
TYMS	thymidylate synthase
UPR	unfolded protein response
WEE1	G2 checkpoint kinase
XRCC1	X-ray repair cross-complementing protein 1
